# Associations between Work Environment and Psychological Distress after a Workplace Terror Attack: The Importance of Role Expectations, Predictability and Leader Support

**DOI:** 10.1371/journal.pone.0119492

**Published:** 2015-03-13

**Authors:** Marianne Skogbrott Birkeland, Morten Birkeland Nielsen, Stein Knardahl, Trond Heir

**Affiliations:** 1 Norwegian Centre for Violence and Traumatic Stress Studies, Oslo, Norway; 2 National Institute of Occupational Health, Oslo, Norway; 3 Department of psychosocial science, University of Bergen, Bergen, Norway; 4 Institute of Clinical Medicine, Faculty of Medicine, University of Oslo, Oslo, Norway; Scuola Superiore Sant'Anna, ITALY

## Abstract

Experiencing terrorism is associated with high levels of psychological distress among survivors. The aim of the present study was to examine whether work environmental factors such as role clarity and predictability, role conflicts, and leader support may protect against elevated levels of psychological distress after a workplace terrorist attack. Data from approximately 1800 ministerial employees were collected ten months after the 2011 Oslo bombing attack which targeted the Norwegian ministries. The results show that after a traumatic event, lower role conflicts, higher role clarity, higher predictability, and higher leader support were independently associated with lower psychological distress. These findings suggest that the workplace environment may be a facilitator of employees’ mental health after stressful events.

## Introduction

The workplace represents a significant arena in life for most adults. A traumatic event at the workplace may therefore have profound consequences for employees. While an extensive body of empirical evidence have shown that factors in the work environment such as role expectations and leadership are associated with health and well-being among employees in general [1–3], little is known about how work factors can have an impact on the after-effects of a trauma, and the literature on associations between psychosocial work factors and psychological distress among employees who have been exposed to trauma is therefore scarce. The present study sought to add to the understanding of how the work environment influence the health consequences of a traumatic experience: the aim of this study was to determine to what extent work environment factors have impact on level of psychological distress in a civilian sample that have been exposed to a terrorist attack targeting their workplace.

Exposure to a traumatic event may result in elevated levels of psychological distress [4,5]. Experiencing a traumatic event may shatter an employee’s most basic cognitive schemes about the world, other people and ourselves [6]. Furthermore, a traumatic event afflicting a workplace in terror attacks, nature disasters or work accidents, subject the employees to demands on top of carrying out regular work. Therefore, when a traumatic event occurs, psychosocial work factors such as high levels of role clarity, low levels of role conflict, high levels of predictability as well as high levels of leader support provide clear expectations and may act as especially important resources that contribute to rebuilding coherent and consistent beliefs and alleviate psychological distress.

It is well-established that both role ambiguity, i.e., uncertainty about what is expected in the job (ambiguous information), and role conflict, i.e., incompatibility between expectations of the employee, are associated with work strain [7,8]. High levels of role ambiguity and role conflict are also related to high levels of psychological distress i.e. depression and anxiety [9,10]. One of the few studies which has assessed the impact of psychosocial work factors after a traumatic event among civilian employees examined a sample of teachers from an area stricken by a major earthquake [11]. In this study, role conflict was associated with increased levels of burnout which includes emotional exhaustion, cynicism and low personal accomplishment).

Along the same lines, lack of predictability at work, which involves insufficient information about decisions, future developments and changes in work, has been linked to burnout [12], cognitive stress reactions [13] and absence from work [14]. A prospective study of the relationship between a broad set of work factors and psychological distress among employees in wide variety of organizations found that leader support was among the most consistent protective factors [10]. Support from a leader may supply the employees with information and feedback that alleviate psychological distress. Low support from supervisor is associated with a higher risk of developing depression [15]. Furthermore, receiving appropriate supervision and support from an ordinate leader may protect against negative consequences of stressful environments [16,17].

Although there are some studies which have examined correlates of traumatic events in safety critical occupations such as military [17–19], police [20] or ambulance personnel [21], these contexts are inherently different from civilian context, where the employees are not prepared or trained to face potentially traumatic events [22]. Naive workplaces that are thrust into extreme contexts without sufficient training and resources to respond to such events, may experience a more extreme and intense situation [23].

By investigating associations between psychosocial work factors and psychological distress in sample of employees from the Norwegian ministries after the 2011 Oslo bombing attack, this study will add to our understanding of how the workplace influence the outcomes of a traumatic event. The bomb explosion resulted in immense destructions of buildings, work equipment and infrastructure of the Norwegian Ministries and can therefore be considered as a major traumatic event for those involved. Specifically, we will examine to what extent role ambiguity, role conflict, predictability and support from immediate leader are related to levels of psychological distress among employees.

## Methods

### Sample and design

In this study we used a sample of ministerial employees who experienced the 2011 Oslo bombing. This was a terror attack directed towards the Norwegian government. A car bomb explosion in the executive governmental quarter in the city center shattered the governmental buildings, killed eight people and injured 209 more people.

In April 2012, approximately ten months after the 2011 Oslo bombing attack, all the 17 Norwegian ministries were invited to participate in the survey study “Mental health and work environment factors in the aftermath of the Oslo terrorist attack July 22nd, 2011”. Three of the ministries did not adhere to the data collection procedures, and were excluded from the study. All employees in 14 of the 17 Norwegian ministries were invited to participate (N = 3520) and 1970 employees, 56% of the total sample, responded. Among these, there were 1133 women and 837 men. In average, non-responders were slightly younger, and the proportion of women was higher among responders compared non-responders. However, the proportion of employees proximate to the bomb attack was similar among the responders and the nonresponders [24]. This paper is based on a cross-sectional data set. All employees provided written consent, strict procedures were followed to ensure confidentiality and the study was approved by the Regional committees for medical and health research ethics in Norway.

### Measures

Psychological distress was measured by the the10-item version of the Hopkins Symptom Checklist, which measures depression- and anxiety-related symptoms [25,26]. The respondents indicated the relevance of each symptom from “have not experienced this symptom” (1) to “have experienced this symptom very much” (4). Cronbach’s alpha was .92.

Role clarity, role conflicts, predictability, and leader support was measured by scales from the General Nordic questionnaire for psychological and social factors at work (QPS Nordic) [27]. Cronbach’s alphas for role clarity, role conflicts, predictability, and leader support were .80, .66, .58, and .87, respectively.

All the respondents were exposed to a terror attack directed at their workplace, which meet the Criterion A for diagnosing PTSD: “The person experienced, witnessed, or was confronted with an event or events that involved actual or threatened death or serious injury, or a threat to the physical integrity of self or others.” (DSM-IV-TR, 2000). Among the respondents of the study, 207 were present at work in the governmental district during the bomb attack, and were coded as physically proximate to the bomb attack (1), whereas the other 1763 respondents were coded as not physically proximate (0).

### Statistical analyses

All data analyses were performed with Mplus Version 7.11 [28]. To correct for the skewed distributions in psychological distress, maximum likelihood estimation with robust errors (MLR) was applied.

In order to study associations between the independent variables and the outcome variable, stepwise linear regression models were conducted in Mplus 7.11. Role conflict, role clarity, predictability, leader support and age were standardized before the regression analyses. Psychological distress was then regressed on these variables, first one at a time (Model I), and then together (Model II). To test whether proximity to the site hit of the explosion influenced the strengths of the associations between work environment and psychological distress, interaction analyses were undertaken (Model III). Adjustment for proximity, gender, and age were made because these variables have been found to be associated with our variables of interests [4,29] (Model IV).

### Missing data

Most of the missing data was caused by individual survey nonresponse. 1707 individuals provided complete data on all items. Among those who responded, the percent of missing data across all variables ranged from 0% (gender) to 7% (items measuring leader support). Full information maximum likelihood (FIML) estimation with robust standard errors was used to handle missing data. This approach uses all observed information to produce the maximum likelihood estimation of parameters. This is one of the best approaches currently available to handle missing data [30].

## Results

Role conflicts, role clarity, predictability and leader support were correlated, but do not show correlations higher than 0.43 (see Table 1).

**Table 1:** Descriptive data.

	Role conflict	Role clarity	Predictability	Leader support	Psychological distress
Role conflict					
Role clarity	−0.36				
Predictability	−0.30	0.38			
Leader support	−0.40	0.43	0.35		
Psychological distress	0.23	−0.24	−0.23	−0.30	
Means	2.51	3.92	4.17	3.92	1.33
SD	0.71	0.74	0.60	0.87	0.48

Means, standard deviations, and correlations between role conflicts, role clarity, predictability, leader support and psychological distress

High levels of role conflict, low levels of role clarity, high levels of predictability and high levels leader support were associated with high levels of psychological distress (see Table 2). Although strengths of the associations were lower in the adjusted models; role conflicts, role clarity, predictability and leader support exhibited significant independent relationships with psychological distress. Interaction analyses showed that proximity to the bomb attack did not influence the relationships between work environmental factors and psychological distress significantly. Controlling for possible confounders such as age, gender and proximity to the bomb attack produced similar results.

**Table 2:** Associations between work environmental factors and psychological distress.

	Model I Bivariate^1^	Model II multivariate	Model III multivariate	Modell IV multivariate
Role conflict	0.23***	0.09**	0.11***	0.09***
Role clarity	−0.24***	−0.09**	−0.11***	−0.09**
Predictability	−0.23***	−0.11***	−0.07**	−0.12***
Leader support	−0.30***	−0.18***	−0.17***	−0.15***
Proximity			0.16***	0.16***
Role conflict X proximity			−0.05	-
Role clarity X proximity			0.07	-
Predictability X proximity			−0.08	-
Leader support X proximity			−0.02	-
Gender				0.17***
Age				0.04

Standardized estimates of associations between work environmental factors and psychological distress in ministerial employees 10 months after the 2011 Oslo bombing; model I-III with results unadjusted, mutually adjusted and adjusted for gender, age and proximity to the terror attack.

^1^ Pearson correlations

** p < .01

*** p < .001

## Discussion

The principle findings of this study were that low levels of role conflict and high levels of role clarity, predictability and leader support were associated with lower levels of psychological distress in employees 10 months after a terror attack against their workplace.

These findings confirm previous findings of associations between work environmental factors and psychological distress [2,3], and extend on them by providing evidence that work environmental factors may be important for recovery after traumatic events. Being exposed to a trauma may produce confusion, feelings of unsafety and anxiety. Clear role expectations, predictability, and leader support at work may increase feelings of safety, provide better information about what to do, improve the possibility to make decisions within the limitations of the situation, allow planning of work tasks, and facilitate coping with the difficult circumstances, which may protect against experiences of strain.

The work characteristics studied here are different concepts, but do exhibit some co-variation. The associations between work characteristics and psychological distress were weaker when all the work factors were taken into account, reflecting the interrelations within work characteristics. This is not surprising, but reflects an inherent challenge when studying a psychological work environment [13]. Still, all the work environmental factors in our study contributed independently to explain psychological distress. This indicates that the employees may benefit from a variety of different and specific characteristics of the work environment.

Leader support seems to be essential for employees’ psychological well-being. Leader support may also be important for other work environment factors [31]. The association between leader support and psychological distress was approximately as strong as the associations between proximity to the bomb explosion and psychological distress, as well as the association between gender and psychological distress. The strength of this association was noteworthy, given that previous research has found proximity and gender to be among the strongest predictors of psychological distress [4,29,32].

To have a sense control involves the belief that one can effectively shape one’s life by one’s efforts and actions, which is important for high psychological well-being [33]. This is also well acknowledged within the literature on work environment [34]. However, in some situations, it may not be possible or feasible to increase employees’ decision latitude. Instead, it may be possible to increase predictability and make role expectations clear, and this may have positive effects on the employees’ psychological health. For example, a supportive leader may provide the employees with both path-goal clarifying and supportive information [17] that may help employees to cope [35]. Studies of organizations’ responses to terrorism and political violence argues that providing information is beneficial [36] and may even be considered a mental health intervention [37]. This may be especially important when sudden events occur in naive organizations who have low probability for such events, and who were not able to prepare or plan for the event.

The main limitation of the present study is the cross-sectional design which does not allow determination of directionality between the phenomena. Level of psychological distress may also influence how the employees experience their psychosocial environment. Furthermore, a considerable amount of data was missing because of nonresponse, which may bias the results. Furthermore, respondents may have selectively revealed or suppressed information, e.g. over-reported positive characteristics and under-reported negative characteristics of their working environment.

## Conclusion

Our study contributes to the field by showing that after trauma, levels of role clarity, role conflict, predictability and leader support are associated with psychological distress among civilian employees.

The present findings suggest specific work characteristics of significance for employee health, even after a major traumatic event. Providing clearly defined job objectives, high level of predictability, and leader support while eliminating role conflicts, can be regarded as factors that can contribute to employee health and thereby help prevent costs arising from reduced work productivity and workplace absence. Research with a qualitative approach that explores how employees exposed to trauma have experienced their work environment might contribute to further knowledge on this field. In addition, studies that measures work environment and employee health at different time points, as well as longitudinal studies might shed light on how these processes unfold across time.
