# Correction: Associations between Work Environment and Psychological Distress after a Workplace Terror Attack: The Importance of Role Expectations, Predictability and Leader Support

**DOI:** 10.1371/journal.pone.0124849

**Published:** 2015-04-09

**Authors:** Marianne Skogbrott Birkeland, Morten Birkeland Nielsen, Stein Knardahl, Trond Heir

The following funder is missing from the Funding section: The Norwegian Council for Mental Health. The complete, correct funding information is as follows: This project has been financially supported by The Norwegian Council for Mental Health and the Norwegian ExtraFoundation for Health and Rehabilitation through EXTRAfunds. Grant number: 2.13/2/0021. URL: http://www.extrastiftelsen.no. The funders had no role in study design, data collection and analysis, decicion to publish, or preparation of the manuscript.
